# Ultrasound-assisted extraction (UAE) of Jerusalem artichoke tuber bio-active ingredient using optimized conditions of Box-Behnken response surface methodology

**DOI:** 10.1016/j.heliyon.2024.e25645

**Published:** 2024-02-13

**Authors:** Newlove A. Afoakwah, William Tchabo, Patrick Owusu-Ansah

**Affiliations:** a Department of Food Science and Technology, Faculty of Agriculture, Food and Consumer Sciences, University for Development Studies, P. O. Box 1882, Tamale, Ghana; b Department of Food Science and Nutrition, National Advanced School of Agro-Industrial Sciences (ENSAI), University of Ngaoundere, Ngaoundere, Cameroon

**Keywords:** Jerusalem artichokes tuber, Antioxidant action, Phenolics, Ultrasound-assisted extraction, Optimal settings

## Abstract

The method of ultrasound-assisted extraction (UAE) was utilized to extract polyphenols from Jerusalem artichokes tuber (JAT). To determine the ideal values for ultrasound power (UP), extraction time (ET), and temperature (TP), a response surface methodology was utilized. JAT extracts were prepared using UAE and their content of total flavonoids (TFC), total polyphenols (TPC), ferric reducing-antioxidant activity (FRAP), and 2, 2′-diphenyl-1-picrylhydrazyl (DPPH) were determined. Accordingly, optimal settings were obtained where TP = 80.0 °C, ET = 14.99 min, and UP = 99.2 °C. These conditions caused TPC, TFC, FRAP, and % DPPH values to reach 4163.6 mg GAE/kg, 2731.6 mg RE/kg, 2.16 mmol/L, and 85.2% respectively, with general-desirability values of 1.00. In addition, DPPH (R = 0.950) and FRAP (R = 0.962) correlated with TPC, indicating that TPC contributed significantly to antioxidant activity. It was found that UAE extraction yields were higher than conventional extraction yields.

## 1 Introduction

Jerusalem artichoke (JA) belongs to the sunflower species and is widely produced in temperate regions for its tuber, utilized as a root vegetable. The JA plant typically reaches a height of 1.5–3 m, while its tubers measure approximately 7.5–10.0 cm and 3.0–5.0 cm in length and thickness respectively. The tuber of JA contains essential components such as vitamins, minerals, protein, inulin, and polyphenols, including sesquiterpenes and coumarin [1]. The utilization of JA extends to treating diabetes and gastrointestinal disorders [2]. Consuming JA aids in reducing the amount of cholesterol echelons, mitigates the endangerment of cancer of the colon, and contributes to fattiness reduction. It decelerates the assimilation of d-glucose and reduces insulin secretion, offering significant benefits for diabetics and non-diabetics [3]. Additionally, Fructose syrups are sourced from JA, which is used in the food sector, it has been employed for medical purposes as fructans [4].

Furthermore, JA exhibits numerous potential functions, including its role in preventing obesity, diabetes, and bacterial and fungal infections [5]. Because of its claimed health benefits, JAT is commonly used in pickled or vegetable salads in China and other Asian countries. JAT has major industrial and dietary implications, particularly in the food industry. Given its health-promoting characteristics, there is a pressing need to develop new techniques for extracting its phenolic components and fractions. The efficiency of polyphenol extractants and their antioxidant capacities are dependent on the quality of the raw biomass and the extraction procedures used [6].

Recently, alternative methods, such as heating extractions, have been employed for extracting phenol compounds, diverging from traditional approaches like Soxhlet, refluxing, and boiling. Nonetheless, these methods have drawbacks [7]. Recently, a variety of cutting-edge extraction systems have been developed to extract phenolic chemicals from plants [8].

They are UAE [9], pressurized solvent extraction [10], and supercritical fluid extraction [11]. The effectiveness of microwave-assisted extraction procedures is impacted by temperature, time, sample mixture, dielectric characteristics, and reagent type [12]. Conversely, the supercritical extraction technique is one of the most used green extraction methods today, showcasing numerous advantages such as higher extraction yields, enhanced selectivity, improved fractionation capabilities, and reduced environmental impacts when compared to classical or traditional extraction processes [13]. Nevertheless, the main drawback of the supercritical extraction technique lies in its high-pressure requirements, leading to increased operating costs. This is attributed to the heightened safety measures necessary compared to other green extraction techniques [14].

On the other hand, UAE extraction serves as a relatively cheap, straightforward, and effective substitute for the conventional solvent extraction method. The process of ultrasonication makes use of the shear force produced when cavitation bubbles crash as a result of the generation of ultrasonic waves. This phenomenon alters material properties and disrupts plant tissues, leading to an enhanced mass transfer [15]. Furthermore, the mechanical action of ultrasound increases the area of contact between the liquid and solid media, allowing for better solvent infiltration through the tissue. Consequently, the solute quickly diffuses into the solvent from its solid phase. Additionally, UAE has been shown to reduce solvent consumption, boost extraction efficiency, require less power, and entail shorter extraction times [16].

To enhance the extraction settings for ultrasound-assisted extraction, the Box-Behnken response surface methodology (RSM) was utilized. RSM employs quantitative data to assess many parameters and their relationships, thereby statistically optimizing the complexities of the extraction technique and so lessening the sum of tests necessary [7].

The study aimed to assess the optimal extraction parameters for JAT using UAE and to compare the extraction effectiveness and antioxidant capacities of the obtained extracts with those obtained through conventional methods.

## 2 Materials and methods

### 2.1 Materials

The JAT was obtained during the winter season from growers in the Yanchen region of Jiangsu province, China. The JAT were washed upon receipt, placed in ice containers, and frozen within an hour upon arrival at the laboratory. The JAT were subsequently repackaged in polythene bags and stored at −18 °C for approximately six days before experimental studies.

### 2.2 Extraction procedures

#### 2.2.1 Ultrasound assisted extraction (UAE)

JAT was extracted using an ultrasound water bath from Electronic Equipment Co. Ltd., China, with an optimal capacity of 4 L. This ultrasonic bath is equipped with adjustable temperature settings, ultrasound power control, timing functions, and a fixed sonication frequency of 60.0 kHz. A homogenized sample weighing 20 ± 0.001g was placed in a sealed rubber bag, along with 50 mL of 60% ethanol. Following UAE, the solvent-JAT mixture underwent centrifugation at 3500.00 rpm for 30.0 min at 4.0 °C with Avanti J-26 XP (Beckman Coulter, Fullerton, USA). The upper liquid was then concentrated with a rotary evaporator at 35.0 °C to obtain the Jerusalem artichoke tuber extract (JATE). The JATE was dehydrated for 2 h at 105 ± 2 °C, and the dehydrated matter was calculated (AOAC 925.09). Additionally, to prepare it for further analysis, the JATE sample was maintained at −18.0 °C in an inert gas environment after it had been diluted with an ethanol solution to a concentration of 1.0 mg/mL.

#### 2.2.2 Conventional extraction

Two traditional extractions were carried out using a slightly modified approach of Tchone [17], Youdim, McDonald [18].

##### 2.2.2.1 Ethyl acetate–methanol (EA) 1:1(v/v)

The fresh JATs weighing 20 ± 0.001g were homogenized in a blender for 5 min in 30 mL of EA. To ensure complete extraction, the liquidizer beaker was rinsed with an additional 10.0 mL EA (1:1, v/v). After 10.0 min of heating in an aquatic bath at 80 °C, the tuber-solvent combination was pressed. The resulting extract was saved at 4.0 °C. An extra 30.0 mL of extracting solvent was added to the initial filtrate and processed in the same manner as described earlier. The second filtrate underwent a similar treatment, but an extracted solution of 10.0 mL was employed. The extracts obtained from these processes were then combined, following the same pooling procedure as described for UAE.

##### 2.2.2.2 Acetone (40 % v/v), methanol (40 % v/v), water (20 % v/v) and formic acid (AC) (0.1 % v/v)

A 20 ± 0.001g sample of JAT was combined with 50 mL AC mixture. This mixture was homogenized for 5 min and then filtered using a filter cloth to separate the extract. The JATE was then obtained using the same retrieval procedure as outlined for UAE.

#### 2.2.3 Design of experiment and analysis of data

Using JAT extract (JAE), a 3-level, 3-factor Box-Behnken model (BBD) was utilized to estimate the consequence of UAE on TPC, TFC, DPPH, and FRAP. Extraction temperature (Z_1_), extraction duration (Z_2_), and the power of ultrasound (Z_3_) were selected as independent factors after the first extraction using single-factor testing **(**Table 1**)**. The BBD studies' findings **(**Table 2**)** were analyzed using the multiple regression equation (1).

(1) $$W=Y_{0}+\sum_{i=1}^{3}Y_{i}Z_{i}+\sum_{i=1}^{3}Y_{ii}Z_{i\;}^{2}+\sum_{i=1}^{2}\sum_{j=i+1}^{3}Y_{ij}Z_{i}Z_{j}$$

Where W (responses), Y0 (coefficients-constant of the intercept), Y*i* (linear)*,* Y*ii* (quadratic), and Y*i*j (interaction terms), while Zi and Z*j* explains the independent-factors representing temperature (Z_1_), extraction duration (Z_2_), and ultrasound power (Z_3_). All experiments were conducted in triplicate, with averages recorded. Version 8.0 of the Design Expert (Stat-Ease, Minneapolis, USA) was used for the arithmetical evaluation. For determining the accuracy of the model, a level of significance of 5% Fisher's F-assessment method was applied. To be more precise, a strong correlation is defined by the model's coefficient determining factor (R^2^), which should be greater than 0.8 for the model to be classified as predictive. For the fitness of the model to be evaluated, the variance analysis (ANOVA) was conducted at a level of significance of 95% confidence. In the absence of fit, P > 0.05 is considered significant, suggesting that the model is adequate.

**Table 1:** Independent variables and their levels were used for Box-Behnken design.

Independent variables, unit	Symbols	Levels		
	Z	−1	0	1
Temperature, T_P_ (°C)	$Z_{1}$	40	60	80
Extraction time, _E_T (Min)	$Z_{2}$	5	10	15
Ultrasound power, U_P_ (Watt)	$Z_{3}$	50	100	150

**Table 2:** Box–Behnken design and the observed responses ox–and observed responses.

Runs	Variables			Responses			
	T_P_ (°C)	_E_T (min)	U_P_ (watt)	Phytochemical compounds			
	$Z_{1}$	$Z_{2}$	*Z*_3_	TPC (mg GAE/kg)	TFC (mg RE/kg)	DPPH (%)	FRAP (mmol/L)
1^a^	60.0	10.00	100.00	3577.83	655.45	72.16	1.85
2	40.0	15.00	100.00	3402.16	1348.30	69.79	1.76
3	80.0	15.00	100.00	4163.27	2532.61	85.14	2.15
4	60.0	5.00	150.00	3611.62	550.59	72.84	1.86
5	60.0	15.00	150.00	3000.00	1577.83	48.17	1.27
6	60.0	5.00	50.00	2795.91	618.71	38.73	1.09
7^a^	60.0	10.00	100.00	3506.12	688.58	70.16	1.79
8	80.0	5.00	100.00	3918.37	644.39	77.86	1.99
9^a^	60.0	10.00	100.00	3478.14	654.46	70.15	1.79
10	80.0	10.00	150.00	3856.23	1971.44	78.95	2.02
11^a^	60.0	10.00	100.00	3612.23	570.61	72.85	1.87
12	40.0	10.00	150.00	3811.74	760.21	78.05	1.96
13	40.0	5.00	100.00	3716.21	1547.46	73.78	1.91
14^a^	60.0	10.00	100.00	3476.31	557.59	70.11	1.79
15	80.0	10.00	50.00	3820.92	1669.46	75.89	1.94
16	60.0	15.00	50.00	3306.75	1214.83	66.69	1.71
17	40.0	10.00	50.00	3306.12	676.58	66.68	1.67

Temperature (T_P_), Extraction time (_E_T), Ultrasound power (U_P_), Total phenolics content (TPC), Total flavonoids content (TFC), 2, 2′-diphenyl-1-picrylhydrazyl (DPPH) and Ferric reducing ability potential (FRAP).

a Central points (used to determine the experimental error).

### 2.3 Analytical methods

#### 2.3.1 Total polyphenol content (TPC) determination

The method outlined by Hossain and Al-Raqmi [19] was applied to estimate the TPC. In short, 0.25 mL of FC reagent was combined with 0.5 mL of the resolubilized extract. Following 2 min of mixing in a vortex mixer, 0.75 mL of a 5% w/v sodium carbonate solution was added after 5 min. After that, the samples were kept for 2 h at room temperature. The results were then expressed in mg GAE/kg as an absorbance test at 765 nm, which was performed using a UV–Vis Spectrophotometer 9600 (Shanghai, China).

#### 2.3.2 Total flavonoid content (TFC) determination

The Hossain, Al-Raqmi technique [19] was used to calculate the TFC. 1.5 mL of NaNO_2_ (0.05% w/v) was added after the resolubilized extract of 0.5 mL and 2 mL of purified water were combined. 6 min were spent incubating the mixture. After adding 1 mL of 1 M NaOH the mixture was left for half an hour at room temperature. the absorbance at 510 nm was quantified with a UV–Vis Spectrophotometer 9600 (Shanghai, China). The mg RE/kg results were reported.

#### 2.3.3 Determination of antioxidant activity

##### 2.3.3.1 DPPH activity assay

The ability of JATE to neutralize free radicals using DPPH was assessed using the Carmona-Jiménez and García-Moreno method [20]. 0.5 mL of the resolubilized extract was combined with 0.1 mM of DPPH radicals in an aqueous solution. After 30 min, a UV–Vis Spectrophotometer 9600 (Shanghai, China) was used to measure the absorbance at 517 nm. Using equation (2), DPPH (%) inhibitory capacity was calculates.

(2) $$\text{Inhibition}\;\left(\%\right)=\left[\left(P_{0}\;–\;P_{1}\right)/P_{0}\right]\times 100$$

Where, P_0_ is the absorbance of the control, and P_1_ is the absorbance of the sample.

##### 2.3.3.2 FRAP technique

The FRAP reagent was created by mixing TPTZ (10 mmol/l), acetate buffer (0.3 mol/L), and FeCl_3_·6H_2_O (20 mmol/L) in a 1:10:1 ratio. Following the addition of 100 μL of JATE to 3.0 mL of FRAP reagent, the mixture was allowed to incubate for 30 min at 37 °C in the dark. The absorbance was then measured at 595 nm using a UV–Vis Spectrophotometer 9600 (Shanghai, China). The results are given in millimoles of Fe2^+^/liter, with ferrous sulphate serving as reference [21].

## 3 Results and discussion

**Fig. 1:**
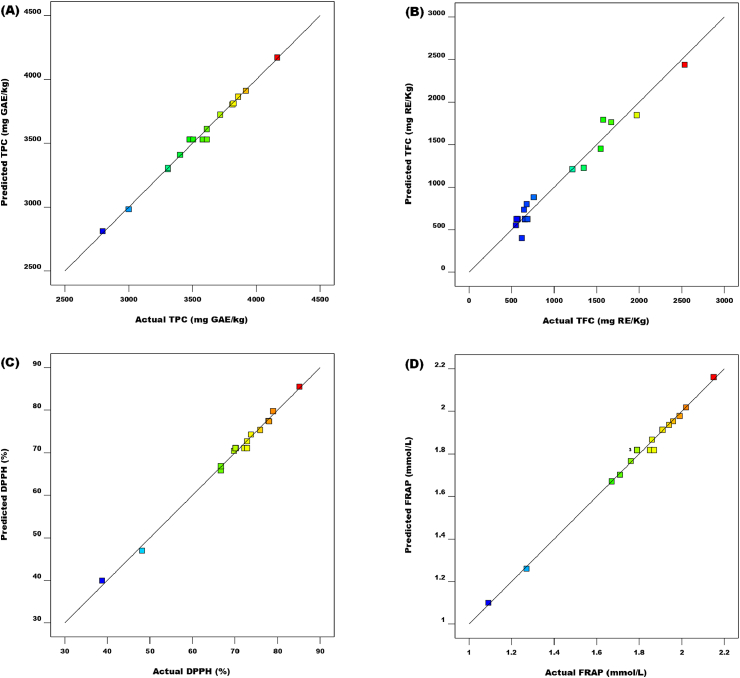
Predicted versus actual plot of (A) total phenolic content; (B) total flavonoid content (C); DPPH radical scavenging activity and (D) Ferric reducing antioxidant power (FRAP).

A total of seventeen (17) experimental runs, each with five (5) duplications at primal points were conducted to evaluate the exact error. The satisfactoriness output model showed that the quadratic function model's "adjusted-R^2^" and "predicted-R^2^" estimates were higher than 0.9804 (Table 3). This suggests that over 98.04% of the entire variance in the yield could be ascribed to the investigated study factors. Furthermore, the variance analysis (ANOVA) presented in Table 4 disclosed that the quadratic model was exceedingly significant at P < 0.0001 for the estimation of phenolics from JATE. Moreover, the model's adequacy fitness demonstrated a strong relationship between the predicted and actual results (Fig. 1[A-D]).

**Table 3:** Summary of the statistical analysis of the tested models.

Source	Sum of squares	DF	Mean square	F value	Prob > F	Std. Dev.	R^2^	Adjusted R^2^	Predicted R^2^	PRESS	Remarks
TPC (mg GAE/kg)											
Mean	2.143E+008	1	2.143E+008								
Linear	2.977E+005	3	99234.34	0.85	0.4890	340.82	0.1647	−0.0281	−0.7393	3.144E+006	
2FI	6.022E+005	3	2.007E+005	2.21	0.1496	301.29	0.4978	0.1965	−1.5417	4.595E+006	
Quadratic	8.923E+005	3	2.974E+005	134.81	<0.0001	46.97	0.9915	0.9805	0.9846	27781.78	Suggested
Cubic	252.83	3	84.28	0.022	0.9948	61.63	0.9916	0.9664			Aliased
Residual	15191.35	4	3797.84								
Total	2.161E+008	17	1.271E+007								
TFC (mg RE/kg)											
Mean	1.957E+007	1	1.957E+007								
Linear	3.731E+006	3	1.244E+006	7.81	0.0031	399.14	0.6431	0.5607	0.4215	3.357E+006	
2FI	78157.78	3	26052.59	0.13	0.9396	446.42	0.6565	0.4505	−0.0635	6.171E+006	
Quadratic	1.976E+006	3	6.588E+005	277.64	<0.0001	48.71	0.9971	0.9935	0.9874	73187.34	Suggested
Cubic	3271.70	3	1090.57	0.33	0.8073	57.74	0.9977	0.9908			Aliased
Residual	13337.69	4	3334.42								
Total	2.537E+007	17	1.492E+006								
DPPH (%)											
Mean	83023.91	1	83023.91								
Linear	175.11	3	58.37	0.42	0.7418	11.79	0.0883	−0.1220	−0.9014	3768.63	
2FI	804.38	3	268.13	2.67	0.1041	10.01	0.4942	0.1907	−1.5700	5093.81	
Quadratic	995.23	3	331.74	317.80	<0.0001	1.02	0.9963	0.9916	0.9918	16.34	Suggested
Cubic	0.34	3	0.11	0.065	0.9755	1.32	0.9965	0.9859			Aliased
Residual	6.97	4	1.74								
Total	85005.94	17	5000.35								
FRAP (mmol/L)											
Mean	54.57	1	54.57								
Linear	0.11	3	0.037	0.50	0.6893	0.27	0.1033	−0.1036	−0.8693	2.02	
2FI	0.43	3	0.14	2.65	0.1059	0.23	0.5006	0.2009	−1.5339	2.74	
Quadratic	0.54	3	0.18	257.60	<0.0001	0.026	0.9955	0.9898	0.9892	0.012	Suggested
Cubic	2.809E-004	3	9.365E-005	0.082	0.9663	0.034	0.9958	0.9831			Aliased
Residual	4.567E-003	4	1.142E-003								
Total	55.66	17	3.27

**Table 4:** ANOVA and regression coefficients of the second-order polynomial model for the response variables (actual values).

Source	DF	TPC (mg GAE/kg)			TFC (mg RE/kg)			DPPH (%)			FRAP (mmol/L)		
		Coefficient	Sum of squares	*P*-Value	Coefficient	Sum of squares	*P*-Value	Coefficient	Sum of squares	*P*-Value	Coefficient	Sum of squares	*P*-Value
Model	9	3530.13	1.792E+006	<0.0001*	625.34	5.786E+006	<0.0001*	71.09	1974.72	<0.0001*	1.82	1.08	<0.0001*
Linear													
_Z1_ (Temp)	1	139.78	1.563E+005	<0.0001*	536.44	2.302E+006	<0.0001*	2.67	57.17	0.0001*	0.079	0.050	<0.0001*
_Z2_ (T_E_)	1	−21.24	3609.84	*0.2416*	414.05	1.372E+006	<0.0001*	0.82	5.40	0.0571	4.015E-003	1.289E-004	0.6791
Z_3_ (M_P_)	1	131.24	1.378E+005	<0.0001*	85.06	57886.27	0.0017*	3.75	112.54	<0.0001*	0.087	0.061	<0.0001*
Interaction													
Z_1_Z_2_	1	240.82	2.320E+005	<0.0001*	70.31	19774.37	0.0234*	4.86	94.37	<0.0001*	0.12	*0.055*	<0.0001*
Z_1_Z_3_	1	−117.58	55297.87	0.0016 *	54.59	11919.34	0.0600	−2.08	17.29	0.0047*	−0.051	*0.010*	0.006*
Z_2_Z_3_	1	−280.62	3.150E+005	<0.0001*	107.78	46464.07	0.0031*	−13.16	692.72	<0.0001*	−0.30	*0.36*	<0.0001*
Quadratic													
Z_11_	1	395.03	6.570E+005	<0.0001*	585.89	1.445E+006	<0.0001*	11.92	598.17	<0.0001*	0.28	*0.32*	<0.0001*
Z_22_	1	−125.15	65950.20	<0.0009*	306.96	3.967E+005	<0.0001*	−6.36	170.56	<0.0001*	−0.14	*0.083*	<0.0001*
Z_33_	1	−226.40	2.158E+005	<0.0001*	58.19	14257.89	0.0440	−8.11	277.25	<0.0001*	−0.20	*0.16*	<0.0001*
Residual	7		15444.18			16609.39			7.31			4.848E-003	
Lack of fit	3		252.83	0.9948		3271.70	0.8073		0.34	0.9755		*2.809E-004*	0.9663
Pure error	4		15191.35			13337.69			6.97			*4.567E-003*	
Total	16		1.808E+006			5.803E+006			1982.03			1.08	
$R^{2}$		0.9915			0.9971			0.9963			0.9955		
Adj-$R^{2}$		0.9805			0.9935			0.9916			0.9898		
C.V. %		1.32			4.54			1.46			1.47		
Adequate precision		38.068			53.139			58.854			52.721		

Total phenolics content (TPC), Total flavonoids content (TFC), 2, 2′-diphenyl-1-picrylhydrazyl (DPPH) Ferric reducing ability potential (FRAP), and Degree of freedom (DF) * indicate 5% significance level.

The least square technique helped in the determination of the interaction, quadratic and linear terms of the model representing Z_1_Z_2_, Z_1_Z_3,_ and Z_2_Z_3_; Z_11_, Z_22,_ and Z_33_; and Z_1,_ Z_2_, Z_3_ respectively. The significance of Z_1_Z_2_, Z_1_Z_3,_ and Z_2_Z_3_; Z_11_, Z_22,_ and Z_33_; and Z_1,_ Z_2_, Z_3_ coefficient effects on the response was examined by ANOVA. As a result of the P-values **(**Table 4**)**, significance levels for each factor were determined. Further, the model's lack of a fit proved to be inconsequential at P > 0.05, meaning that the fitted data' prediction was reliable.

### 3.1 Response surface analysis of total phenolic content

**Fig. 2:**
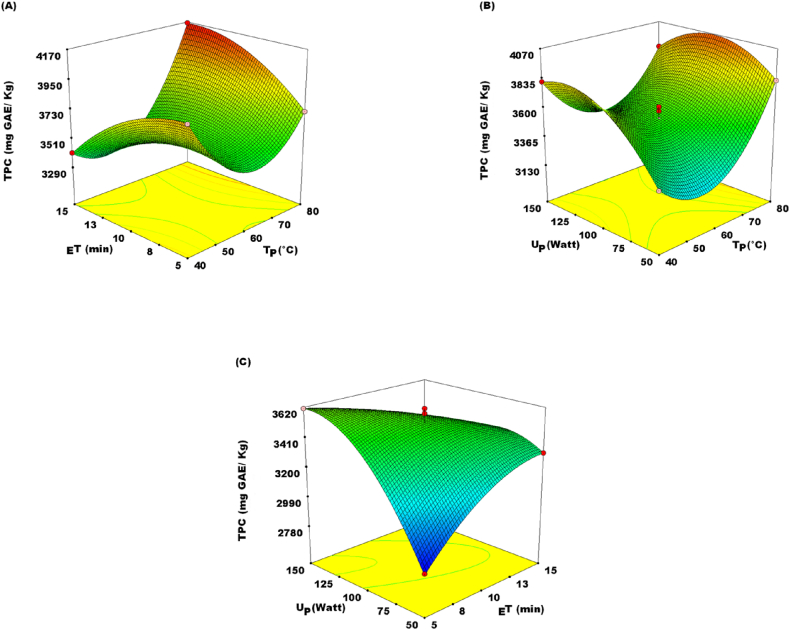
Response surface plots showing the effect of (A) extraction time (_E_T) and temperature (T_P_); (B) ultrasound power (U_P_) and temperature (T_P_); (C) ultrasound power (U_P_) and extraction time (_E_T) on total phenolics content (TPC).

TPC significance level of *P* < 0.05 influenced the quadratics (Z_11_, Z_22,_ and Z_33_), linear (X_1_ and X_3_), and interactive (Z_1_Z_2_, Z_1_Z_3,_ and Z_2_Z_3_) terms of this study (Table 4). The best parameters for the extraction of phenolic chemicals, as projected by equation (3) were found to be: temperature (TP) = 40.11 °C, ultrasound power (UP) = 147.95 W, and extraction period (ET) = 5.85 min. Grounded on these optimized settings, the TPC reached 4164.97 mg GAE/kg. However, as shown in Fig. 2A and B, the TPC of the JA extract increased significantly with a rise in TP from 60.0 to 80.0 °C. These outcomes suggest that the process of extracting phenolics from JATE may be more favorable at moderate temperature ranges. At lower temperatures, the TPC was affected, leading to a decrease. This result concurs with Spigno, Tramelli [22] who concluded that a temperature of 60 °C favors phenolic yield than at lower temperatures. Mild temperatures (60–80 °C) may have the effect of softening plant tissues, hydrolyzing the bonds of bound phenolic compounds, weakening cell wall integrity, and enhancing the solubility of phenolics [22]. Hence, more phenolics may dole out to the extracting solvent, and enhance the extraction of TPC. Also, mild TP might increase the extraction output, due to these properties: diffusivity, viscosity, surface tension, and solubility [23]. Ultrasound can expedite hydration, swelling, and the enlargement of pores in plant cells, thereby enhancing the turnover of solute components from plants resources to the extracting liquid [24]. Therefore, increasing in U_P_ led to an increase in TPC (Fig. 2. C).

The derangement of plant compartments, facilitated by a micro jet following the collapse of cavitation bubbles, could potentially increase the solvent diffusion rate into the structural material of the plant [24]. Moreover, the total phenolics levels showed increased with an increase in U_P_ [25]. As depicted in Table 4, the quadratic terms of Z_22_ and Z_33_ hurt TPC. This means that with further increase in _E_T and U_P_ would trigger a decrease in TPC. Also, the interaction terms Z_1_Z_3_ and Z_2_Z_3_ revealed a negative (p < 0.005) effect on TPC. The explanation for this may be linked to longer _E_T and increasing levels of UP-inducing polyphenol disintegration [26]. The reduction in TPC at higher power may perhaps be associated with the oxidation response, caused by the free radicals (•OH) interaction created due to sonication [27].

The predictive equation for the TPC was considered using the second-order equation depicted in equation (3).

(3) $$Y_{\text{TPC}}=3530.13+139.78Z_{1}+131.24Z_{2}+240.82\;Z_{3}\;–\;117.5Z_{1}Z_{2}\;-\;280.62Z_{1}^{2}+395.03Z_{2}^{2}\;-125.15Z_{3}^{2}\;-\;226.40\;Z_{4}^{2}$$

### 3.2 Response surface analysis of total flavonoid content

**Fig. 3:**
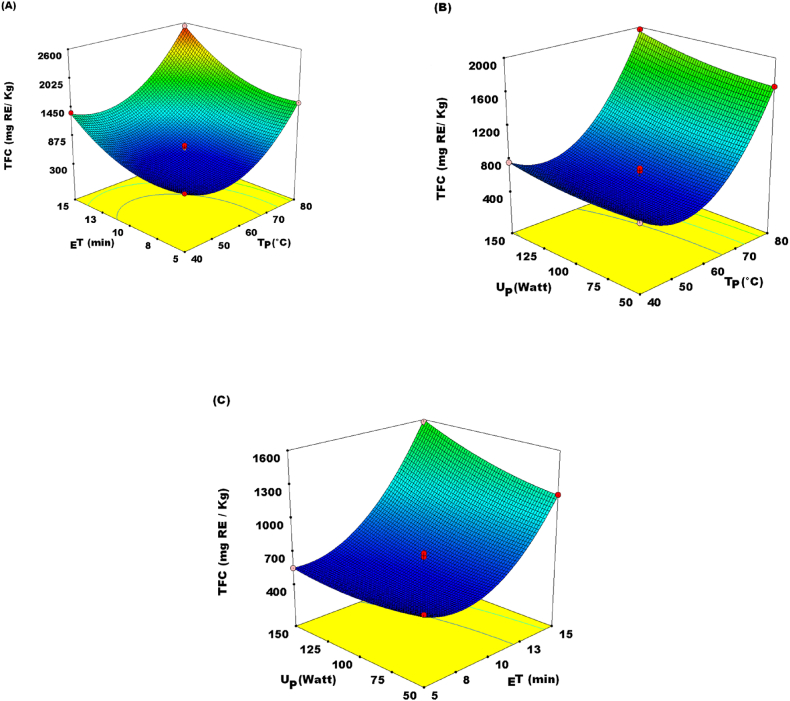
Response surface plots showing the effect of (A) extraction time (_E_T) and temperature (T_P_); (B) ultrasound power (U_P_) and temperature (T_P_); (C) ultrasound power (U_P_) and extraction time (_E_T) on total flavonoid content (TFC).

_E_T, T_P_, and U_p_ parameters, and their effect on UAE for the extraction of TFC were studied. Table 4 portrays the linearity of T_P_ (*P* < 0.05), _E_T at *P* < 0.05, and U_P_ at *P* < 0.05, which demonstrated a substantial enhancement in the TFC of the JATE. As well, Z_1_Z_2_ and Z_2_Z_3_ were demonstrated to be significant at *P* < 0.05 on TFC. In addition, Z_11_ and Z_22_ as quadratic terms established a substantial outcome at *P* < 0.05. Employing the examined indexes, the TFC of JATE ranged from 550.59 to 2532.61 mg RE/kg (Table 2). The ideal parameters stipulated for TFC, computed from equation (4) were achieved at T_P_ = 78.93 °C, U_P_ = 128.41 W, and _E_T = 14.75 min. The predicted flavonoid content was found to be 2546.61 mg RE/kg. As seen in Fig. 3A the TFC of the JATE was enhanced linearly with an elevated T_P_ and _E_T. Increasing T_P_ might boost solvent extraction by accelerating the coefficients of flavonoid solubility and diffusion [28]. Additionally, at advanced temperatures, flavonoid particles may speedily diffuse from the cells into the extraction medium [28]. Furthermore, a longer duration of _E_T at optimum U_P_ caused the liberation of TFC (Fig. 3C). As stated by Zhou [29] U_P_ demonstrated a substantial outcome when extracting total flavonoids. Besides, from modest to a higher U_P_, a significant (*P* < 0.005) linear effect was observed for flavonoid extractions. For any _E_T and T_P_, the investigated total flavonoid values increased with increasing U_P_ (Fig. 3B). A regression model for BBD was built using the ANOVA of the study results and is shown in equation (4).

(4) $$Y_{\text{TFC}}=625.34+536.44Z_{1}+414.05Z_{2}+85.06Z_{3}+70.31Z_{1}Z_{2\;}+107.78\;Z_{1}Z_{2\;}+585.89Z_{1}^{2}+306.96Z_{2}^{2}$$

### 3.3 Analysis of the response surface for antioxidant activity

Numerous studies have provided evidence that polyphenols possess preventive properties against various health conditions, including cardiovascular diseases, cancer, obesity, and diabetes [30,31]. Polyphenols' antioxidant properties shed light on their relationship to health benefits. Polyphenols combat free radicals by using their intrinsic redox characteristics. They operate as scavengers, breaking the chain reactions resulting in lipid peroxidation. They produce stable phenolic-radical compounds by providing electrons for reactive free radical species in the body, therefore balancing potentially damaging chain reactions in cellular activities. It is believed that this antioxidant activity helps explain how polyphenols shield the body against illnesses linked to oxidative stress [32]. As molecular barriers, antioxidants slow down the Fenton process and prevent reactive hydroxyl radicals from oxidizing molecules [32].

**Fig. 4:**
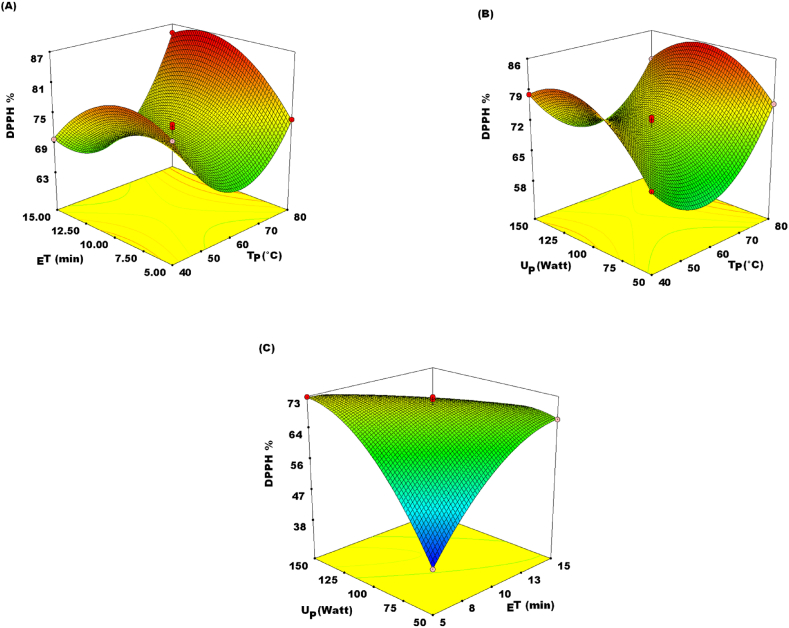
Response surface plots showing the effect of (A) extraction time (_E_T) and temperature (T_P_); (B) ultrasound power (U_P_) and temperature (T_P_); (C) ultrasound power (U_P_) and extraction time (_E_T) on DPPH (2,2-Diphenyl-1-picrylhydrazyl) radical scavenging activity.

**Fig. 5:**
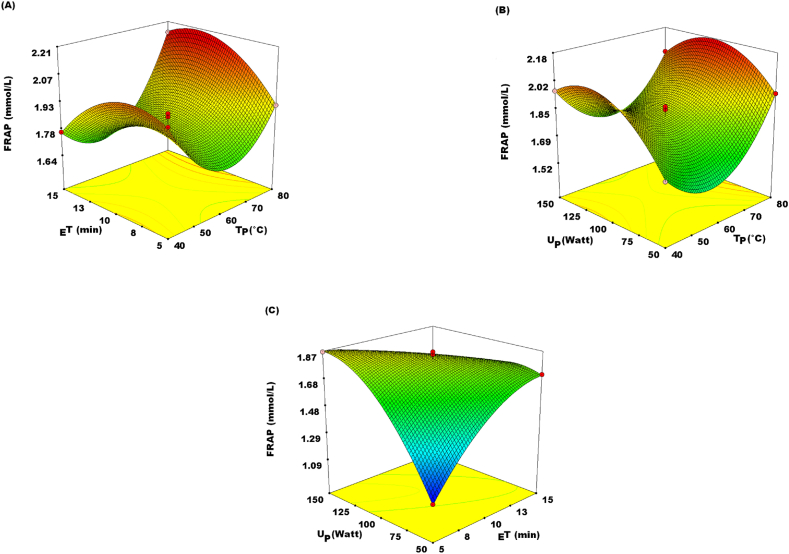
Response surface plots showing the effect of (A) extraction time (_E_T) and temperature (T_P_); (B) ultrasound power U_P_) and temperature (T_P_); (C) ultrasound power (U_P_) and extraction time (_E_T) on Ferric reducing antioxidant power (FRAP).

For DPPH and FRAP, the total antioxidant activity (TAA) result of JATE utilizing UAE ranged from 38.73 to 85.14% and 1.09–2.15 mmol/L, respectively **(**Table 2**)**. These are contingent on the range of selected experimental settings. Z_11_, Z_22_, and Z_33_, the quadratic terms, Z_1_ and Z_3_, linear terms and interaction terms, Z_1_Z_2_, Z_1_Z_3_, and Z_2_Z_3_, were shown to be significant at P < 0.05 for both the FRAP and DPPH **(**Table 4**)**. Notably, the TAC value rose with rising TP, U_P,_ and _E_T up to 80.0 °C, 150 W, and 15 min respectively (Fig. 4. A, B&C and Fig. 5. A, B&C). The optimal parameters for % inhibition of DPPH and FRAP value were projected to be attained at T_P_ 40.24 °C; and 78.05 °C. _E_T at 6.40 min; 13.04 min. U_P_ at 126.86 W; 90.53 W. Equations 5 and 6's centering on the second-order polynomial model yielded a FRAP of 2.15 mmol/L and an estimated % DPPH inhibition of 85.51%. The TAC showed similar results to the TPC in terms of the quadratic impact (P < 0.05) Z_11_, Z_22_, and Z_33_, and the linear effect Z_1_Z_3_ (P < 0.05), as well as interaction effect Z_1_Z_2_, Z_1_Z_3_, and Z_2_Z_3_ at P < 0.05.

**Fig. 6:**
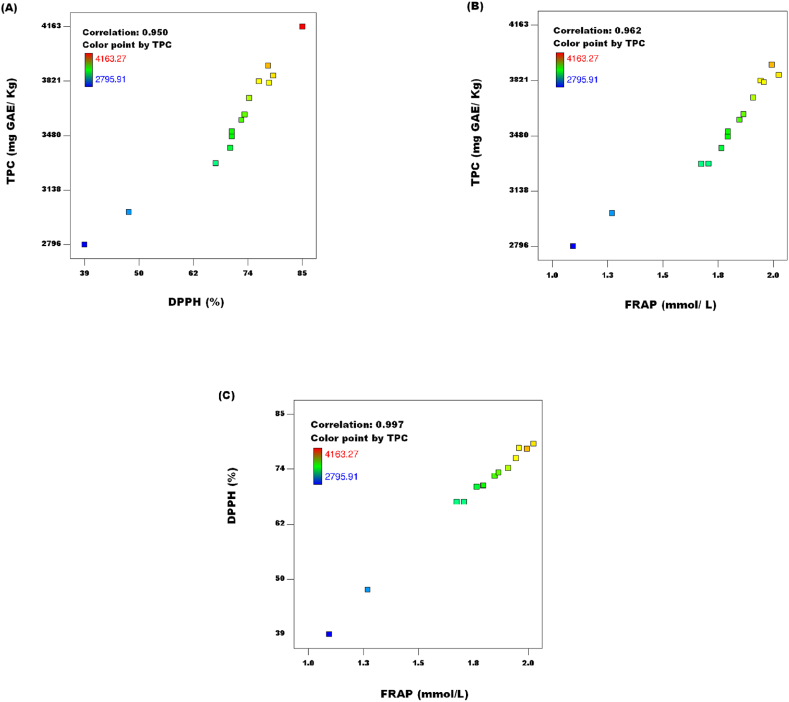
Correlations between responses: (A) total phenolic content (TPC) and DPPH (2,2-Diphenyl-1-picrylhydrazyl) radical scavenging activity; (B) total polyphenol content and Ferric reducing antioxidant power (FRAP); (C) DPPH (2,2-Diphenyl-1-picrylhydrazyl) radical scavenging activity and Ferric reducing antioxidant power (FRAP).

Fig. 6] showed a direct correlation between the TPC in the extracts and the TAA of the JATE. Compared to those of DPPH (R = 0.950) in the JATE (Fig. 6A), FRAP of the JATE had a stronger correlation (R = 0.962) with the TPC (Fig. 6B). This suggested that the main phenolic component in charge of the JATE's total antioxidant activity was TPC. This conclusion is in line with the findings of earlier studies [33,34]. Also, it was found that FRAP corelated at R = 0.997 with DPPH (Fig. 6C). The TAA's second-order polynomial equations (5) and (6) look like this:

(5) $$Y_{\text{DPPH}}=71.09+2.67Z_{1}+3.75Z_{3}+4.86Z_{1}Z_{2}-2.08\;Z_{1}Z_{3}\;-13.16Z_{2}Z_{3}+11.92Z_{1}^{2}-6.36Z_{2}^{2}\;-8.11Z_{3}^{2}$$

(6) $$Y_{\text{FRAP}}=1.82+0.079Z_{1}+0.087Z_{3}+0.12Z_{1}Z_{2}-0.051\;Z_{1}Z_{3}-0.30Z_{2}Z_{3}+0.28Z_{1}^{2}-0.14Z_{2}^{2}-0.20Z_{3}^{2}$$

### 3.4 Optimization of the extraction parameters

**Fig. 7:**
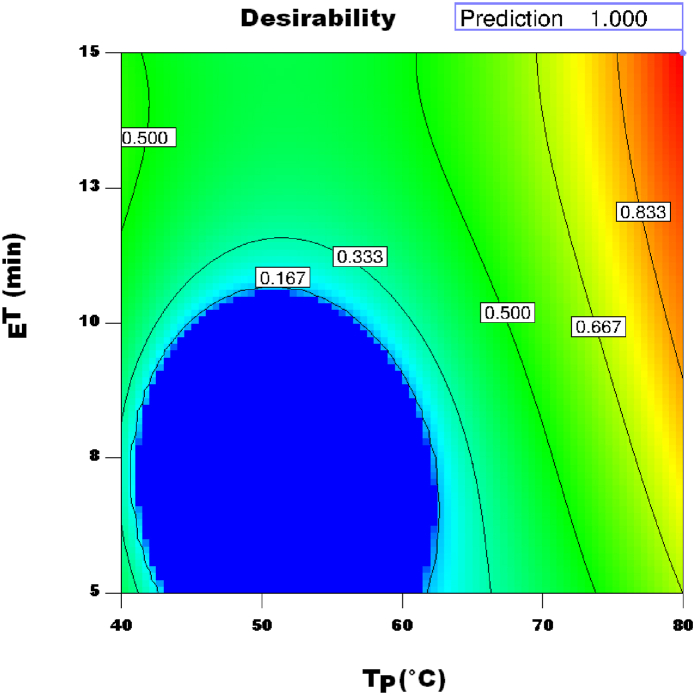
Desirability index plot for optimal extraction conditions of Jerusalem artichoke tuber phenolics.

The chosen optimized parameters for using the UAE extraction method to extract phenolics from JAT were: T_P_ = 80.0 °C, _E_T = 14.99 min, and U_P_ = 99.20 W. The optimal condition was adopted to generate more TPC, TFC, DPPH, and FRAP. The level best values of TPC = 4163.60 mg GAE/kg, TFC = 2731.59 mg RE/kg, DPPH = 85.15%, and FRAP = 2.16 mmol/L were obtained with a general-desirable measure of 1.00 as a result of the ideal conditions chosen (Fig. 7).

### 3.5 Validation of the predictive model

Under ideal conditions of triplicate experiment, a slight adjustment was made to the original experiment to account for the possibility of production. Employing TP of 80.0 °C, ET of 15 min, and UP of 100 W to verify that the model was suitable for forecasting the optimal responses. Under optimal ultrasonic extraction conditions, the JA extract's TPC of 4098.20 ± 199 (mg GAE/kg), TFC of 2704.46 ± 67 (mg RE/kg), DPPH of 84.83 ± 0.43%, and FRAP of 1.89 ± 0.09 mmol/L were detected. The results showed that there was no significant difference (P > 0.05) between the expected and experimental values.

### 3.6 Comparison of the different extraction techniques

**Fig. 8:**
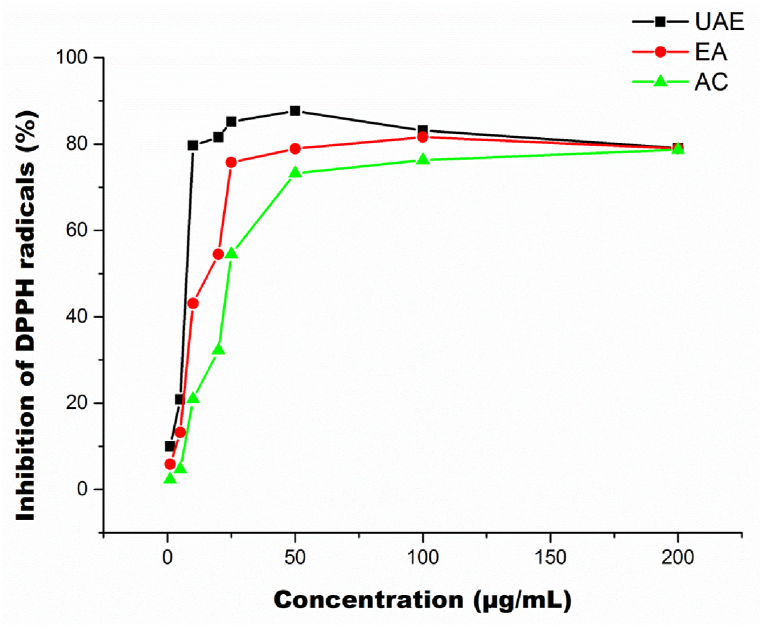
DPPH radicals scavenging activity of extracts obtained by Ultrasound-Assisted Extraction (UAE), Ethyl Acetate- Methanol (EA) extraction, Acetone: Methanol: Formic acid: Water extraction (AC).

The efficiency of UAE was confirmed by conducting two conventional extraction techniques. Consequently, these results were obtained: TPC of the JATE was 2106.23 ± 42 mg GAE/kg, 1231.19 ± 64 mg GAE/Kg, and 4098.20 ± 199 mg GAE/kg for EA extraction, AC and UAE, correspondingly. The findings of the TPC are exhibited as follows UAE > EA > AC extraction. The TPC in the dissimilar extraction techniques varied significantly (*P* < 0.05). The TFC of UAE was 2704.46 ± 67 mg RE/kg, followed by EA extraction (2013.19 ± 49 mg RE/kg) and AC extraction (1145.20 ± 76 mg RE/kg). Concerning the DPPH of the JATE the following was the categorization UAE (84.83 ± 0.43 %) > EA extractions (79.12 ± 0.28%) > AC extraction (61.23 ± 28%). The following was observed for the FRAP of the JATE: UAE (1.89 ± 0.09 mmol/L) > EA extractions (1.09 ± 0.08 mmol/L) > AC extraction (0.76 ± 0.03 mmol/L). Additionally, as illustrated in Fig. 8, the DPPH inhibition capabilities are compared to liquid-extraction procedures between a range of 0–200 μg/mL. As projected, extracts obtained through UAE showcased a superior ability to scavenge free DPPH radicals compared to extracts obtained through conventional extraction techniques. The outcomes showed that when compared to traditional procedures, the UAE was more successful at extracting polyphenols from JATE. A comparable outcome was previously reported by Zhou [29], using UAE. They highlighted that the UAE could extract polyphenols from plant resources in a shorter time compared to conventional techniques. Furthermore, Pingret, Fabiano-Tixier [35] demonstrated a similar result, indicating that UAE was more effective than conventional techniques. According to earlier studies, improved mass transfer of extracts could be the reason for the UAE's high extraction efficiency and consequently increased extraction yield [36].

## 4 Conclusions

This study demonstrated that Jerusalem artichoke tuber (JAT) could be a notable source of phenolics. Second-order mathematical models derived from Box-Behnken response surface methodology explained the observed findings. There was a significant effect of temperature, ultrasound power, and extraction time on TFC in JATE as determined by multiple regression analyses These factors also had an impact on TPC FRAP and DPPH. The optimal parameters for increasing polyphenol content and antioxidant activity were established to be a TP of 80.0 °C, _E_T of 14.99 min, and U_P_ of 99.20 W. Additionally, comparing the conventional extraction method with the UAE method revealed that UAE yielded higher polyphenol contents and antioxidant activities. The efficient and potent characteristics of UAE make it a promising technique for the food industry and the extraction of bioactive from Jerusalem artichokes tuber, offering the potential for higher extraction outputs.

## Funding statement

Funding organizations in the governmental, private, or nonprofit sectors did not provide a specific grant for this study.

## Data availability statement

Data will be made available on request.

## CRediT authorship contribution statement

**Newlove A. Afoakwah:** Supervision, Methodology, Conceptualization. **William Tchabo:** Software, Investigation, Formal analysis. **Patrick Owusu-Ansah:** Data curation.

## Declaration of competing interest

The authors declare the following financial interests/personal relationships which may be considered as potential competing interests:Afoakwah Newlove reports statistical analysis was provided by University for Development Studies. Newlove Afoakwah reports a relationship with Univesity for Development Studies, Tamale, Ghana that includes: non-financial support. Newlove Afoakwah has patent pending to Assignee. Authors are not in any of the editorial board of Heliyon If there are other authors, they declare that they have no known competing financial interests or personal relationships that could have appeared to influence the work reported in this paper.
